# Adaptive sampling during sequencing reveals the origins of the bovine reproductive tract microbiome across reproductive stages and sexes

**DOI:** 10.1038/s41598-022-19022-w

**Published:** 2022-09-05

**Authors:** Chian Teng Ong, Elizabeth M. Ross, Gry Boe-Hansen, Conny Turni, Ben J. Hayes, Geoffry Fordyce, Ala E. Tabor

**Affiliations:** 1 Queensland Alliance for Agriculture and Food Innovation, Centre for Animal Science, The University of Queensland, Brisbane, QLD 4072 Australia; 2 Faculty of Science, School of Veterinary Science, The University of Queensland, Brisbane, QLD 4072 Australia; 3 Faculty of Science, School of Chemistry and Molecular Bioscience, The University of Queensland, Brisbane, QLD 4072 Australia

**Keywords:** Microbial communities, Microbial genetics, Sequencing

## Abstract

Cattle enterprises are one of the major livestock production systems globally and are forecasted to have stable growth in the next decade. To facilitate sustainable live weight production, optimal reproductive performance is essential. Microbial colonisation in the reproductive tract has been demonstrated as one of the factors contributing to bovine reproductive performance. Studies also implied that reproductive metagenomes are different at each stage of the estrous cycle. This study applied Oxford Nanopore Technologies’ adaptive long-read sequencing to profile the bovine reproductive microbiome collected from tropical cattle in northern Queensland, Australia. The microbiome samples were collected from cattle of different sexes, reproductive status and locations to provide a comprehensive view of the bovine reproductive microbiome in northern Australian cattle. Ascomycota, Firmicutes and Proteobacteria were abundant phyla identified in the bovine reproductive metagenomes of Australian cattle regardless of sexes, reproductive status and location. The species level taxonomical investigation suggested that gastrointestinal metagenome and the surrounding environment were potentially the origins of the bovine reproductive metagenome. Functional profiles further affirmed this implication, revealing that the reproductive metagenomes of the prepubertal and postpartum animals were dominated by microorganisms that catabolise dietary polysaccharides as an energy substrate while that of the pregnant animals had the function of harvesting energy from aromatic compounds. Bovine reproductive metagenome investigations can be employed to trace the origins of abnormal metagenomes, which is beneficial for disease prevention and control. Additionally, our results demonstrated different reproductive metagenome diversities between cattle from two different locations. The variation in diversity within one location can serve as the indicator of abnormal reproductive metagenome, but between locations inferences cannot be made. We suggest establishing localised metagenomic indices that can be used to infer abnormal reproductive metagenomes which contribute to abortion or sub-fertility.

## Introduction

### Importance of cattle production

The cattle industries are important elements of global agricultural production. Beef production is projected to increase ~ 5.8% by 2030^1^. Population and income growth have driven global beef consumption, primarily in Asia and the Pacific. Milk consumption is also expected to increase by 1.2% per annum in the next decade as a result of population and income growth^1^. To meet this increasing demand, reproductive performance in cattle needs to be maintained and optimised for production and profitability.

Good reproductive performance is important for optimal production from both beef and dairy cattle^2^. In general cattle have a maximum of one pregnancy and one offspring per year, making bovine reproduction less efficient than other farm animals such as pigs and sheep^3,4^. Any disruption to reproductive performance introduces delays in reproductive processes, and subsequently reduces the live weight production as well as the biological and business efficiency of production^5,6^. To avoid costly reproductive delays, reproductive tract health needs to be kept optimal.

### Microbiome and reproductive performance

The “sterile womb paradigm” suggests that in a healthy pregnancy the womb is free from microorganisms. However, this prevailing hypothesis was challenged by recent reproductive tract microbiome studies^7–9^. The impact of the reproductive tract microbiome on abortion, sub-fertility and infertility remains unclear, and the pathogenicity of the microbiome as well as host immunological responses are the hypothetical determining factors^10,11^.

In cattle, it is common that the uterus is contaminated with a wide range of microbes ascending from the environment or the animal’s skin and faeces^12,13^. The anatomical location of the bovine reproductive tract allows the direct microbial colonisation from the gut through faeces and from soil. Following this colonisation cows can restore their uterus and clear the microorganisms by rapid involution, discharge of the reproductive tract content, as well as activation of immune responses^14,15^. Persistent bacterial infection because of adherence and colonisation of the reproductive tract potentially leads to the development of clinical reproductive diseases if the host defence system is compromised^13,15^. Direct invasion and toxin secretion by the pathogenic microbes impair the reproductive tract tissues, disturb hormonal regulation and induce host immune regulators. These perturbations in the cattle reproductive system render an unfavourable local environments for the transportation of gametes and the viability of the embryo^16^.

Metagenomic studies demonstrated that the commensal microflora of the bovine reproductive tract is dominated by Bacteroidetes, Firmicutes and Proteobacteria, and secondarily by Actinobacteria, Fusobacteria and Tenericutes^9^. Studies of the bovine reproductive tract microbiome have previously not identified an association between any specific microbial species and the development of reproductive diseases or infertility^17–19^. This is despite known pathogenic microbial species that infect the reproductive tract^12,20^. However, bovine reproductive diseases have been associated with the increased abundances of bacteria from phyla Bacteroidetes and Fusobacteria as well as the decrease of microbiome diversity, which could be used to inform diagnoses.

This study aimed to investigate the determining factors of bovine reproductive metagenomes by comparing the differences of the bovine reproductive metagenomes collected from animals of different sexes, reproductive status and different cattle stations. We employed Oxford Nanopore Technologies (ONT) long-read adaptive sampling to profile the bovine reproductive metagenomes collected in this study to enable unbiased taxonomic and functional characterisation, while reducing host DNA contamination.

## Materials and methods

### Ethics declarations

All procedures involving animal use were approved under Animal Ethics Approval AE30009 by the Animal Ethics Committee of the University of Queensland (UQ). All protocols in this study were performed in accordance with the UQ Animal Ethics Committee approved standard operating procedures (SOPs) for Companion and Production Animals. The study was carried out in compliance with the ARRIVE guidelines.

### Sample collection and extraction

Samples were collected from two cattle properties, which were approximately 200 km apart, in Northern Queensland by an experienced veterinarian. The cattle breed in Station A and B were Crossbred and Droughtmaster respectively and the *Bos indicus* content of the herds in these two stations were 49 and 54% respectively (Supplementary file Appendix 1). In total, four hundred and fifty-two samples were collected from 2018 to 2021.

Samples from bulls were labelled as “Male”. For heifers and cows, the stages of pregnancy and reproductive status were determined using transrectal ultrasound as previously detailed^21^. Briefly: prepubertal were heifers which did not have a corpus luteum (CL); cycling referred to animals that had a CL present but no fetus or embryo was detected; pregnant were the animals in which a fetus or embryo was detected; postpartum were cows which were known to have delivered a calf in the past 12 months (Supplementary file Appendix 2).

The preputial samples were collected by inserting the Tricamper™ (DAF Queensland, Australia) sampling tool into the prepuce. The Tricamper™ was moved back and forth to scrape across the preputial mucosa and the surface of the penis. For vaginal samples, the Tricamper™ was inserted into the vagina and moved back and forth with the leading edge in contact with the anterior walls of the vagina to collect the sample. The vaginal or preputial sample was immediately preserved in a 10 mL tube preloaded with 5 mL phosphate-buffered saline (PBS) by excising the head of the Tricamper™ device.

The samples were kept chilled from collection and through delivery, which normally takes 1 to 2 days, and were processed within 6 h upon arrival to the laboratory. Each sample was first vortexed for 15 s and followed by an additional 15 s vortex after the Tricamper™ head was removed from the tube. The vaginal mucus from the tubes were then transferred into a sterile 15 mL tube. The vaginal samples were pelleted by centrifugation and the supernatant was removed. The samples were extracted using QIAGEN DNeasy Blood & Tissue kit (QIAGEN, Hilden, Germany) according to the manufacturer’s instruction for Gram-positive bacteria. There were not enough replicates for both the cycling females in Station A and the male samples from Station B due to poor quality and quantity of the extracted DNA samples; therefore, these two groups of animals were not included in the downstream analyses. In total, 37 metagenome samples were sequenced in this study and the sequence data were deposited to NCBI database with Accession Number from SAMN26105035 to SAMN26105071 (Supplementary file Appendix 2).

### Oxford Nanopore Technologies adaptive sampling

Adaptive sampling was conducted as described previously^22^. Firstly, the quantity of extracted DNA was measured using Qubit™ 4 fluorometer (Invitrogen™) with Qubit™ dsDNA Broad Range assay kit (Invitrogen™). The extracted gDNA were examined using the Pippin Pulse (Sage Science) pulsed-field electrophoresis gel to determine the size and integrity. Libraries for ONT adaptive sampling were prepared using the ONT SQK-LSK109 kit (ONT, Cambridge, UK) according to the manufacturer’s instructions. Briefly, 48 uL of the extracted DNA was added to the end-repair reaction mix. Then, adapters were ligated to the end-repaired DNA. The ligated DNA library was loaded to be sequenced on an individual MinION flowcells FLO-MIN106D (ONT, Cambridge, UK). Each time, three to five libraries were running concurrently on the ONT GridION Mk1 sequencer with software MinKNOW version 21.05.8 (ONT, Cambridge, UK). The adaptive sampling mode was applied to deplete genomes of both ARS-UCD1.2 *Bos taurus* genome (GCA_002263795.2) and Brahman genome^23^. Each sample was sequenced for 24 h and the raw data was transferred to Linux system for base calling using Guppy version 5.0.11.

The base-called reads were examined using NanoPlot 1.3.0^24^. Porechop 0.2.4^25^ was performed to remove the adapters on the long reads while NanoFilt 2.7.0^24^ was conducted to remove reads which were lesser than 5 in quality score. To ensure complete removal of the bovine reads, the remaining long reads were re-mapped against the ARS-UCD1.2 *Bos taurus* genome (GCA_002263795.2) and Brahman genome^23^ using Minimap2 2.17 (r941)^26^. The unmapped reads were selected for downstream analysis.

### Contig construction

Flye 2.8.3-b1725^27^ with the ‘*–meta*’ setting was used to assemble the metagenomic data with uneven coverage. A co-assembly was also constructed using the reads from all samples with Flye 2.8.3-b1725^27^. Coverage depth calculation was obtained using the jgi_summarize_bam_contig_depths script from Metabat2 v2.15^28^.

### Metagenomic classification

Read-based taxonomic profiling was performed using Kraken v2.1.2^29^. A customised database was constructed with the build script provided by Kraken2^29^ to ensure a more targeted and efficient search for the organisms in the metagenome samples collected for this study. The customised database used in this study was built with the complete genomes of archaea, bacteria and fungi, which were downloaded from NCBI RefSeq^30^ with their low complexity sequences masked. Bracken v2.6.2^31^ was performed using the output generated from Kraken v2.1.2^29^ to estimate the abundances of the organisms in the metagenome samples. The downstream bioinformatic analyses and visualisation of the results were conducted on R studio^32^ with R packages including vegan 2.5.7^33^, phyloseq 1.34.0^34^, DEseq2 1.30.1^35^, dplyr 1.0.7^36^ and ggplot2 3.3.5^37^.

The pipeline MetaErg 1.2.0^38^ was performed to functionally annotate the assembled contigs of each sample. Briefly, the predicted ORFs were subjected to HMMs profile similarity searches or DIAMOND (double index alignment of next-generation sequencing data) searches against several databases, including Pfam-A^39^, TIGRFAM^40^, FOAM^41^, metabolic-hmms^42^, *cas*genes.hmm^43^ and SwissProt^44^. Mapping files generated from searches against SwissProt, FOAM and TIGRFAMs databases were incorporated in MinPath^45^ to infer to KEGG^46^ and MetaCyc^47^ metabolic pathways. Bioinformatic analyses and visualisation of the outputs were performed on R studio^32^ with R packages including^48^ edgeR 3.32.1^48^ and clusterProfiler 3.18.1^49^.

### Ethical approval

All procedures involving animal use were approved under Animal Ethics Approval AE30009 by the Animal Ethics Committee of the University of Queensland (UQ). All protocols in this study were performed in accordance with the UQ Animal Ethics Committee approved standard operating procedures (SOPs) for Companion and Production Animals.

## Results

In total, ONT adaptive sampling yielded an average of 12.67 Gb and 6,299,065 Kbp of raw data per sample (Supplementary file Appendix 3). After host contamination removal and quality filtering, there was an average of 417, 843 Kbp of data, in each of the bovine metagenome samples (Supplementary file Appendix 4 and 5).

**Figure 1:**
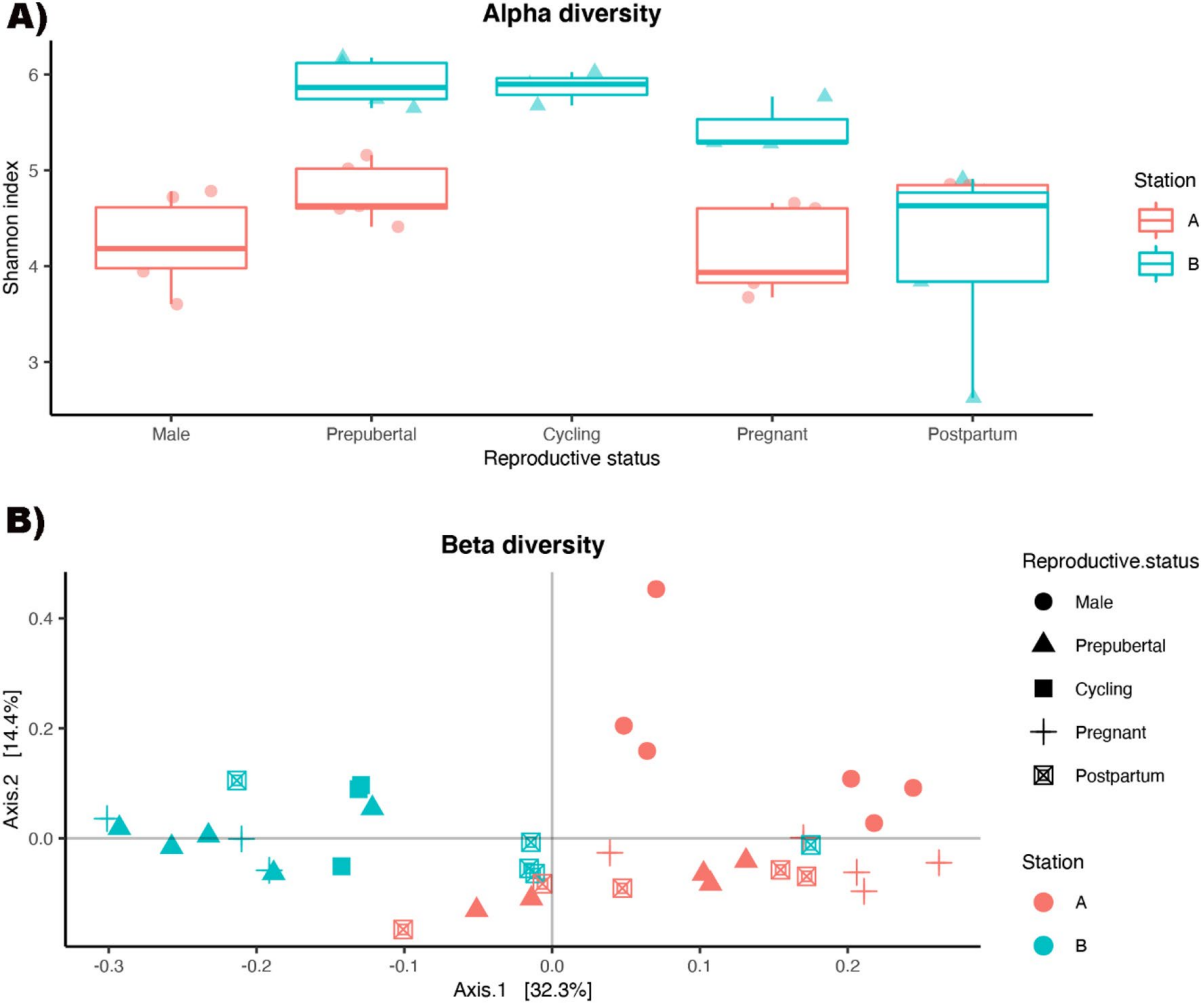
(**A**) Alpha diversity and (**B**) beta diversity of the reproductive tract metagenomes collected from Station A (red) and Station B (blue). The shapes represent animals of different sexes or reproductive status: circle represents Male, triangle represents prepubertal animals, square represents cycling animals, cross represents pregnant animals and square cross represents postpartum animals.

The alpha diversity, represented by Shannon index, indicated that the bovine reproductive metagenomes from different representative groups in Station A had similar alpha diversities, regardless sexes and reproductive stages (ANOVA, *P* = 0.129) (Fig. 1). On the other hand, the bovine reproductive metagenomes posed significantly different (ANOVA, *P* = 2.0 × 10^–3^) diversity in Station B. Particularly, the reproductive metagenome of postpartum animals had significantly lower diversity than prepubertal, cycling and pregnant animals (*T*-test, *P* = 4.43 × 10^−4^, 1.64 × 10^−3^ and 1.0 × 10^−2^ respectively). In general, the bovine reproductive metagenomes in Station B were significantly more diverse (*T*-test, *P* = 7.12 × 10^–4^) than those in Station A.

The beta diversity represented by principal coordinate analysis ordination (PCoA) of the Bray–Curtis dissimilarity matrix demonstrated that there were significant dissimilarities (PERMANOVA, *P* = 9 × 10^–4^) between the bovine reproductive tract metagenomes collected from Station A and B. Within Station A, the dissimilarity between bovine male and female reproductive tract metagenomes was also significant (PERMANOVA, *P* = 2 × 10^–3^). Nonetheless, in either of the station, the bovine female vaginal metagenomes were not significantly different between different reproductive stages (Permutation test, *P* > 0.05).

**Figure 2:**
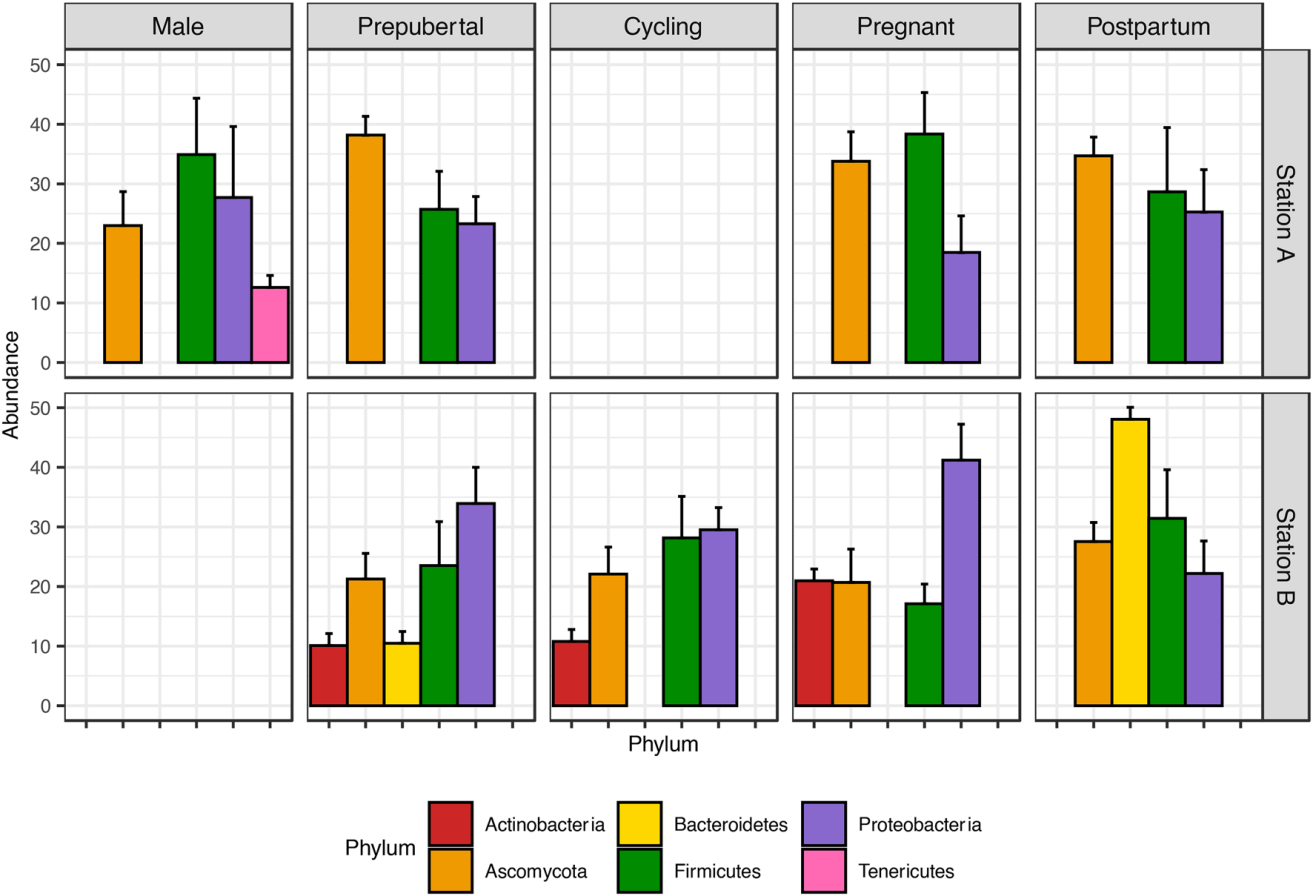
Phyla with more than 10% of abundance in the bovine reproductive metagenomes collected from Station A and Station B. Empty panels indicate no animal was sampled in that category and empty columns indicate phylum less than 10%.

Figure 2 depicted the abundant phylum, including those with more than 5% abundances in the bovine reproductive tract metagenome. Ascomycota, Firmicutes and Proteobacteria each constituted more than 20% of the bovine reproductive tract metagenome, regardless of sexes or reproductive stage. Bacteroidetes was only abundant in the bovine vaginal metagenomes collected from Station B while phylum Fusobacteria was only abundant in the postpartum bovine vaginal metagenome collected from Station B. Actinobacteria and Tenericutes were identified in all samples, however their abundances were not consistently higher than 5% at different stations, sexes and reproductive stages.

**Figure 3:**
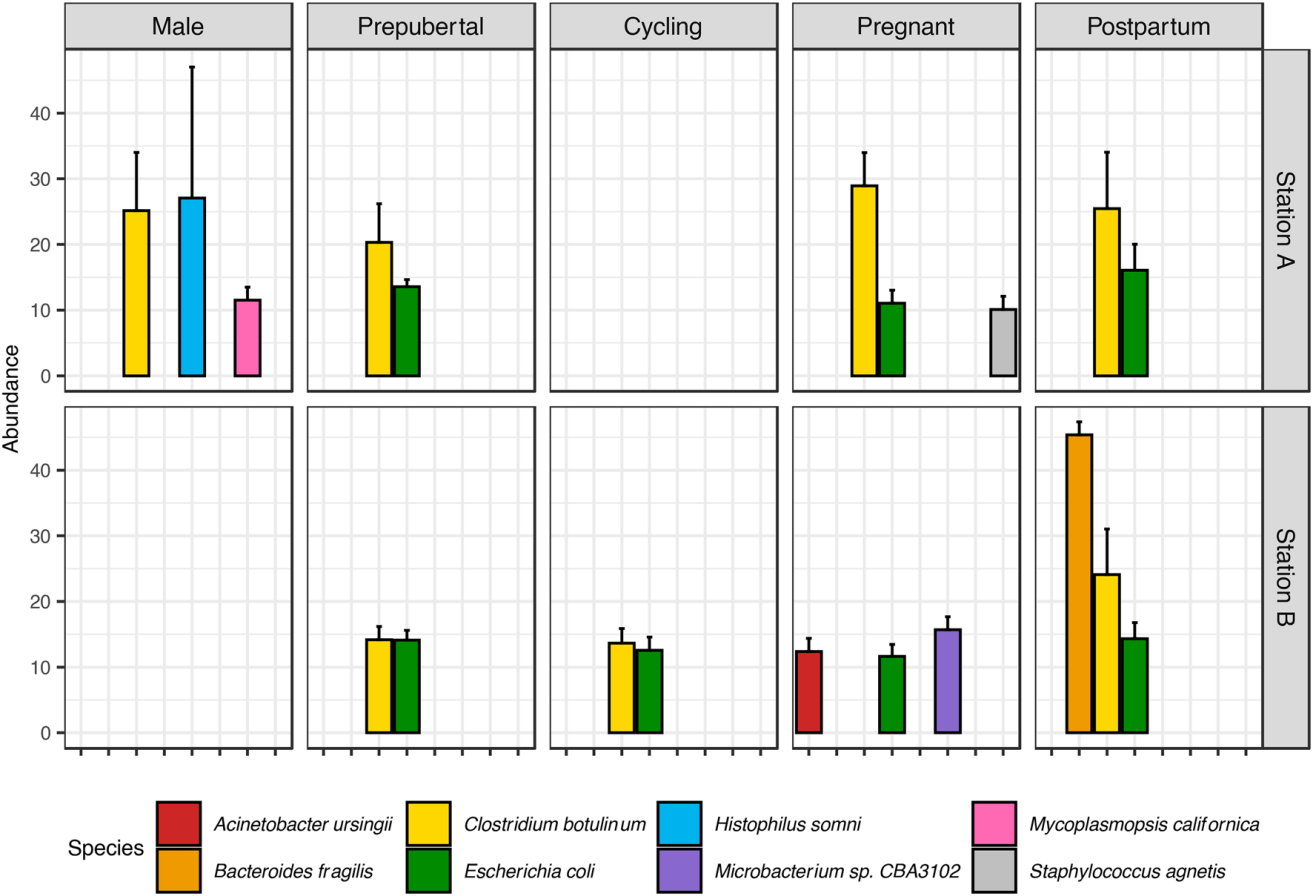
Species with more than 10% of abundance in the bovine reproductive metagenomes collected from Station A and Station B. Empty panels indicate no animal was sampled in that category and empty columns indicate species less than 10%.

At the species level, all the top abundant species were bacteria (Fig. 3). In Station A, *Clostridium botulinum* was commonly abundant regardless of sexes and reproductive status. *Escherichia coli* was only commonly abundant in the female reproductive metagenomes, while *Histophilus somni* (previously *Haemophilus somnus*) and *Mycoplasmopsis californica* were the abundant species in the preputial samples only. *Staphylococcus agnetis* was abundant in pregnant animals but not in prepubertal and postpartum animals. Similarly, *E. coli* was commonly abundant in all female reproductive metagenomes in Station B. Except for Pregnant animals, *C. botulinum* was the common abundant species in the vagina metagenomes collected from Station B. Instead, the Pregnant animals in Station B had more than 10% of *Acinetobacter ursingii* and *Microbacterium sp.* CBA3102. *Bacteroides fragilis* was the most abundant species in the postpartum vaginal metagenomes collected from Station B.

**Figure 4:**
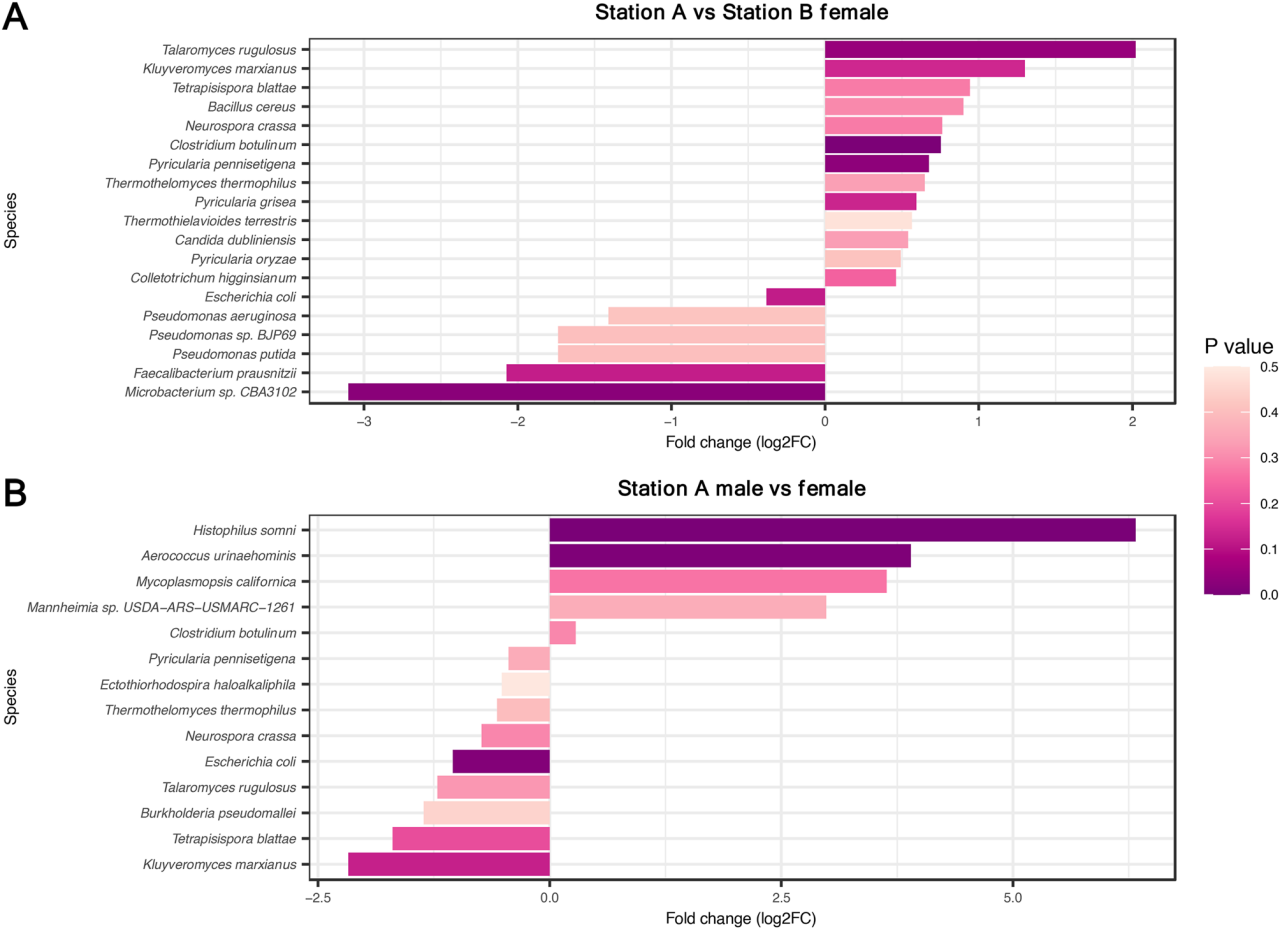
Microbes log_2_ fold change comparing (**A**) Station A against Station B and (**B**) male against female in Station A.

We compared and identified the species which had significantly different abundances between Station A and Station B (Fig. 4A) and between the two sexes in Station A (Fig. 4B). The species which were more significantly abundant in Station A were *Clostridium botulinum* (Wald test, *P* = 2.57 × 10^−3^), *Pycularia pennisetigena* (Wald test, *P* = 2.48 × 10^−2^) and *Talaromyces rugulosus* (Wald test, *P* = 4.83 × 10^–2^), while the species which was more significantly abundant in Station B was *Microbacterium sp.* CBA3102 (Wald test, *P* = 8 × 10^−3^). Within Station A, metagenome samples collected from bull prepuce had a significantly higher abundance of *Histophilus somni* (Wald test, *P* = 4.21 × 10^−7^) and *Aerococcus urinaehominis* (Wald test, *P* = 8.36 × 10^−3^). The female vaginal metagenomes had significantly higher abundances of *Escherichia coli* (Wald test, *P* = 1.47 × 10^−2^) than the bull preputial samples.

In the multidimensional scaling (MDS) analysis, which depicted the dissimilarity based on the leading log_2_ fold change of the functional annotations, it was observed that most of the bovine vaginal metagenomes collected from Station B were clustered together (Supplementary file Appendix 6). Similarly, the MDS analysis demonstrated that there were higher dissimilarities between bovine reproductive tract metagenomes collected from different sexes.

**Figure 5:**
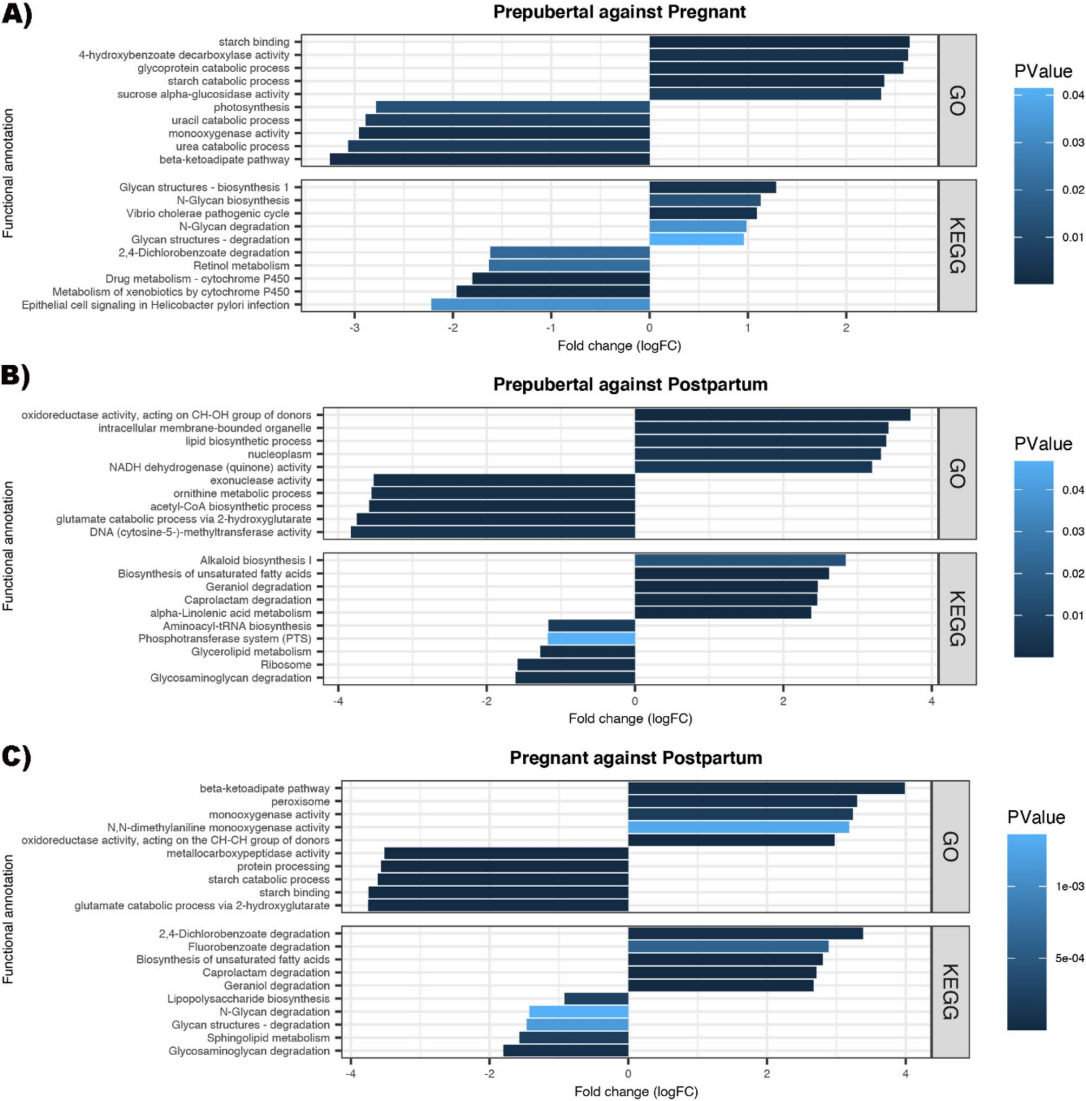
Top 5 significantly more and less expressed GO terms and KEGG pathways of the bovine reproductive metagenome comparing (**A**) prepubertal and pregnant animals, (**B**) prepubertal and postpartum animals and (**C**) pregnant and postpartum animals.

Comparisons were conducted for the bovine vaginal metagenome collected from prepubertal, pregnant and postpartum groups. The top 5 GO terms and KEGG pathways, which were significantly enriched when compared with the other reproductive stage, were demonstrated (Fig. 5). There was a significantly lower expression of “Starch binding”, “Starch catabolic process”, “N-Glycan degradation” and “Glycan structures—degradation” in the bovine vaginal metagenome collected from Pregnant animals as compared to prepubertal and postpartum animals. Additionally, Pregnant animals also have greater expression of “beta-ketoadipate pathway” and “monooxygenase activity” than prepubertal and postpartum animals. Functional annotations, including “Glutamate catabolic process via 2-hydroxyglutarate” and “Glycosaminoglycan degradation” were significantly greater while “Biosynthesis of unsaturated fatty acids”, “Caprolactam degradation” and “Geraniol degradation” were significantly less expressed in the bovine vaginal metagenome of the postpartum animals.

## Discussion

Our study corresponded to the previous investigations which proposed that bovine reproductive tract metagenomes originate from the environment and their gastrointestinal tracts^50^. In addition to Firmicutes and Proteobacteria, Ascomycota was also identified as a commonly abundant phyla in our study. However, there was no association of reproductive diseases with a single microbe. Our results demonstrated different diversities, both in the number of species (alpha diversity) and change in species (beta diversity), in the bovine reproductive metagenomes collected from two cattle stations. In general, the animals from Station B had a greater number of species in their bovine reproductive metagenomes in comparison to the animals from Station A and the species identified in the two stations had significantly low level of similarity. Cluster analysis suggested that the species diversity in the bovine reproductive metagenome was not solely determined by the reproductive status. We concluded that the location of the animals, and the environment that they are exposed to, has a larger effect on the reproductive tract than reproductive status.

Most of bovine reproductive metagenome studies have previously been conducted using 16S amplicon short-read sequencing technology, which is limited to identifying genomes which expressed the 16S ribosomal RNA gene and do not inform the functional aspects of the microbiome^51^. In our study, we adopted ONT long-read technology for adaptive metagenomic sequencing, which reduced the host contamination and enabled non-targeted metagenome identification. The longer sequences also provided more information for taxonomical and functional annotations of the metagenome^22^. The unbiased long-read sequencing approach employed in this study returned an extensive metagenome profile with greater resolution without the hassle of sequencing the sample with different amplicon targets. For example, our study identified Ascomycota as the commensal phyla besides Firmicutes and Proteobacteria, which were commonly reported in the previous studies^9^. In the past, Ascomycota has only been reported in the bovine reproductive metagenome sequenced using amplicon D1/D2^52,53^. Even though ONT adaptive sampling unbiasedly recovered a more extensive metagenomic profile^22^, further investigation shall be conducted to determine and compare its accuracy in recalling metagenomic signatures and relative abundances with other sequencing technologies.

*Escherichia coli* was identified commonly in the female reproductive metagenomes collected in this study. The previous studies used standard culture technique have identified *E. coli* as the causative agents of bovine reproductive diseases^12^. Nevertheless, the recent metagenomic studies have identified *E. coli* from the reproductive tract of both healthy cows and cows with reproductive diseases^54–57^. Host–pathogen interaction studies also suggested that both the host immunity and pathogenicity of the *E. coli* strains determined the potential for disease onset^20,54,58^. Therefore, *E. coli* is not a good indicator of bovine reproductive diseases such as metritis since it can be present in a healthy and functional reproductive tract as a commensal organism.

The most abundant species in male preputial sample was *Histophilus somni*, which was not reported as an abundant species in the female reproductive metagenomes. *Histophilus somni* is a commensal member in the bovine respiratory and reproductive tracts^59,60^. Studies have shown that inoculation of *H. somni* into the reproductive tracts of pregnant cows resulted in different degree of infections, however abortion was not observed nor was *H. somni* recovered from the calves^60^. Nonetheless, abortion and systemic immune response were also observed in both experimental inoculation and with natural colonisation of *H. somni*^61–64^. The inconsistent results of the *H. somni* infection studies support the notion that opportunistic infections caused by *H. somni* can also be determined by a combination of factors, including compromised host immunity, intercurrent infection and severe stress^59,65^. Therefore, identification of a single microbe, for example *E. coli* or *H. somni*, is not a good indicator of disease.

The metagenomic profiles collected in this study signified the colonisation of environmental and gastrointestinal microbiome in the bovine reproductive tract. In our study, the two beef cattle breeds had similar *Bos taurus indicus* content and breed was less likely to contribute to the reproductive metagenomes as suggested by previous study which demonstrated the great similarity between the vaginal microbiomes of two breeds of *Bos taurus indicus*, Gyr and Nellore^53^. Instead, transmission of prevalent microbes into the reproductive tract is potentially from faeces or soil due to the close anatomical proximity^52,66^ especially when the physical barrier, for example cervix, is compromised^67^, or via blood from the gastrointestinal tract^68^. The dominant genus under Ascomycota in our samples was *Pyricularia*, which was first recorded in 1999 to cause blight of buffelgrass (*Cenchrus ciliaris*) in Queensland Australia^69^ and recognised as one of the potential causative agents responsible for the pasture dieback in Australia^70,71^. The most prevalent genus from Ascomycota reported in previous studies was *Mycosphaerella*, which is a common fungus in soil^52,53^. Like *Mycosphaerella* in the previous studies, in this study *Pyricularia* potentially gained access to the bovine reproductive tract directly from the environment or indirectly from the gastrointestinal tract after being ingested with the pasture feed.

The other commonly abundant species identified in this study was *C. botulinum*. The dominance of genus *Clostridium* in the healthy bovine reproductive metagenome has been reported previously^52,53,66,72,73^. The spores of *C. botulinum* are commonly identified in soil or in the guts of healthy animals in tropical environments^74^. Spore germination of *C. botulinum* happens in an anaerobic environment will induce neurotoxin production and lead to a paralysis disease called botulism^75^. Several botulism outbreaks in Queensland Australia have been recorded since 1922^76,77^. Since *Clostridium* is widely prevalent in the sewage, faeces, soil and animal carcasses, they potentially gained access to the bovine vaginas because of the close anatomical proximity of the reproductive tract to the environment^66,78,79^.

There were greater levels of microbial fermentation activities of complex carbohydrates and host-derived glycans, in the bovine reproductive metagenomes of prepubertal and postpartum animals than pregnant animals. Microbial fermentation of starch is the symbiont mechanism of ruminant gut microorganisms to facilitate the digestion and energy release from complex polysaccharides^80^. Degradation of glycan and glycosaminoglycan, which forms the epithelial lining of the gastrointestinal and reproductive tracts^81,82^, can also be manipulated by commensal organisms to access nutrients as well as by disease-causing pathogens to deplete the protective host mucus layers^83,84^. Greater levels of microbial fermentation activities in the vaginas of the prepubertal and postpartum animals indicated the vaginal colonisation by the microorganisms originated from the gastrointestinal tract. A decrease of glycan degradation in the reproductive metagenome function of the pregnant animals is likely an essential process for the decidual reaction during pregnancy, allowing the thickening of the mucosal lining for fetus implantation^85^.

There were higher levels of caprolactam and geraniol degradation activities in the bovine reproductive metagenomes of prepubertal and pregnant animals than in postpartum animals. Caprolactam and geraniol degradations are part of the sub-network that is significantly enriched in the soil rhizosphere microbiome. Additionally, the pregnant animals’ vaginal microbiome demonstrated a significantly higher level of beta-ketoadipate pathway and monooxygenase activity, which are essential for environmental microorganisms to derive energy and growth substrates by degrading aromatic compounds^86^. Aromatic compounds are common in feedlots and paddock challenged with different degrees of environmental challenges depending on the moisture and manure management. Hence, the dominating microorganism detected in the vagina of the prepubertal and pregnant animals originate from the soil.

Although the reproductive statuses of the animals were assessed by an experienced veterinarian with ultrasound scanning, the “Cycling” animals was potentially misidentified as “Prepubertal” due to the difficulty to detect the corpus luteum during estrous cycle. Nonetheless, the reproductive metagenomes of “Prepubertal” and “Cycling” animals were not significantly different in both alpha and beta diversities. Additionally, the reproductive metagenome samples from the “Postpartum” animals were collected during the routine mustering of the cattle farm, which was between 6 and 12 months from the previous mustering. The long-time interval had potentially caused the reproductive tract to recover from their postpartum infections and developed varying reproductive physiologies. Even though the alpha diversity of the preputial metagenome was not significantly different from the vaginal metagenome collected from the same site, there was a significant dissimilarity in their beta diversity. No study has previously been conducted to compare the bovine male and female reproductive metagenomes. The change in species diversity can be attributed to the anatomical locations as well as the functionalities of the metagenomes^87,88^.

In this study, the animals in each station portrayed similar diversity levels in their reproductive tract metagenomes regardless of reproductive status, except for the reproductive tract metagenomes from the postpartum animals collected from Station B which had significantly lower diversity than the other groups of females.

Station B had low diversity and increased abundances of Bacteroidetes and Fusobacteria in the postpartum reproductive metagenomes. Station B anecdotally had a lower pregnancy rate than Station B (data not shown). Increases of Bacteroidetes and Fusobacteria in bovine reproductive metagenomes have been associated with the development of reproductive diseases^9^. Failure to restore from postpartum contamination may have led to dysbiosis and dominances of the opportunistic microbes, which are often associated with bovine reproductive infections^72,89,90^.

## Conclusion

In this paper, we demonstrated that ONT adaptive sampling methods enabled an unbiased and more accurate profiling of the bovine reproductive microbiomes. Both the taxonomical and functional profiles in this study reinforced the potential of environmental and gut microbiome colonisation in the bovine reproductive tract. We also validated that the presence of potentially pathogenic species does not indicate suboptimal reproductive health. Instead of low diversity, our results showed that a decrease in diversity together with increases of Bacteroidetes and Fusobacteria abundance are the indications of a suboptimal reproductive metagenome. Therefore, assessments of reproductive microbiome health need to be conducted in the context of what is normal for that location or region and cannot be extrapolated across geographical locations. Since the host-associated microbiomes are an interconnected network of communities that are continually exchanging, instead of separated ecological niches, we recommend that a unique microbiome index for each farm or pasture could be established to indicate the fertility of the herd on a routine basis. Additionally, good hygiene and waste management practices play an essential role in reducing the risks of pathogen colonisation and dysbiosis in the bovine reproductive tracts.

## Supplementary Information

Supplementary Information.

## Data Availability

The datasets generated during the current study are available in the NCBI sequence read archive (SRA) database under BioProject PRJNA808759, BioSamples SAMN26105035 to SAMN26105071.

## Abbreviations

ONT: Oxford Nanopore Technologies
CL: Corpus luteum
PBS: Phosphate-buffered saline
PCoA: Principal coordinate analysis ordination
MDS: Multidimensional scaling
