# Supplementary material for: Adaptive sampling during sequencing reveals the origins of the bovine reproductive tract microbiome across reproductive stages and sexes
Source: Sci Rep. 2022 Sep 5;12:15075. doi: 10.1038/s41598-022-19022-w (PMC9445037; doi:10.1038/s41598-022-19022-w)
Supplement: Supplementary file 1 — Supplementary Information. [file 41598_2022_19022_MOESM1_ESM.docx]

Appendix 1: Breed makeup and *Bos indicus* content (%) of the herds in Station A and Station B.


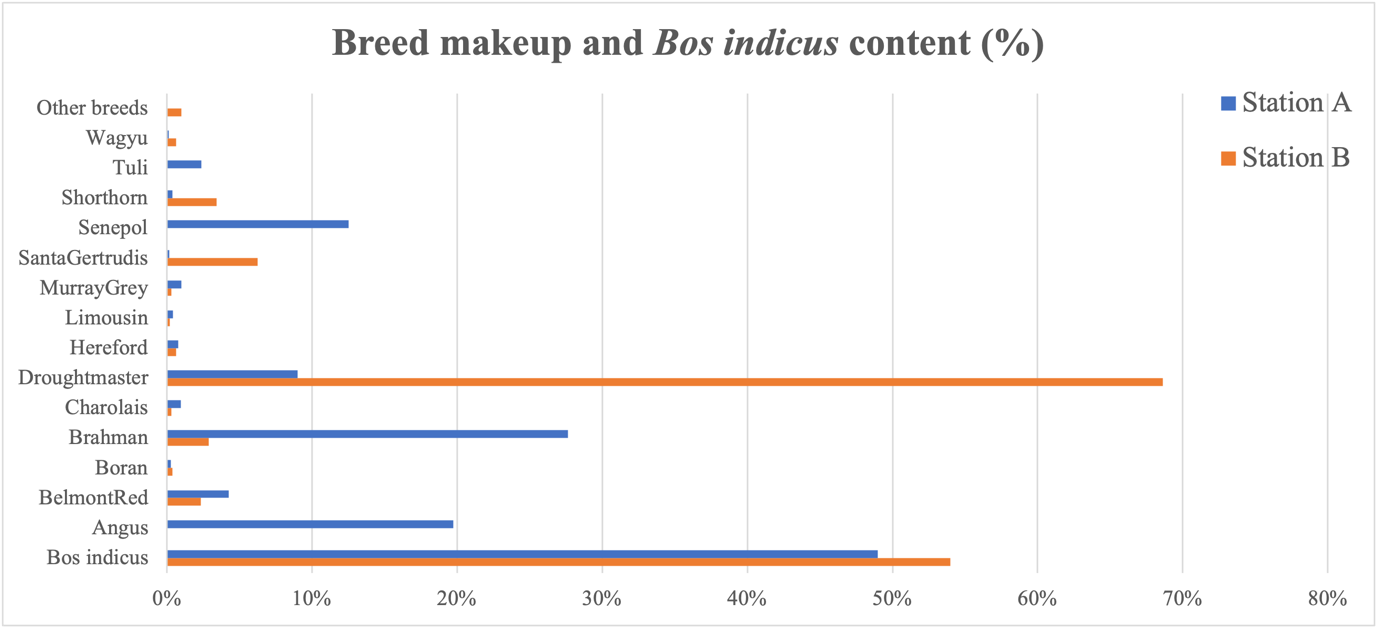


Appendix 2: Thirty-seven samples collected from Station A and Station B.

| ID | Birth year | Station | Gender | Breed | Reproductive status | Sampling year |
| --- | --- | --- | --- | --- | --- | --- |
| 264 | 2017 | Station A | Female | Crossbred | Pregnant | 2018 |
| 265 | 2017 | Station A | Female | Crossbred | Prepubertal | 2018 |
| 267 | 2017 | Station A | Female | Crossbred | Prepubertal | 2018 |
| 270 | 2017 | Station A | Female | Crossbred | Prepubertal | 2018 |
| 271 | 2017 | Station A | Female | Crossbred | Pregnant | 2018 |
| 272 | 2017 | Station A | Female | Crossbred | Pregnant | 2018 |
| 273 | 2017 | Station A | Female | Crossbred | Prepubertal | 2018 |
| 277 | 2017 | Station A | Female | Crossbred | Pregnant | 2018 |
| 278 | 2017 | Station A | Female | Crossbred | Prepubertal | 2018 |
| 280 | 2017 | Station A | Female | Crossbred | Pregnant | 2018 |
| 294 | 2016 | Station A | Male | Crossbred | NA | 2018 |
| 295 | 2016 | Station A | Male | Crossbred | NA | 2018 |
| 299 | 2013 | Station A | Male | Crossbred | NA | 2018 |
| 918 | 2017 | Station B | Female | Droughtmaster | Postpartum | 2021 |
| 924 | 2017 | Station B | Female | Droughtmaster | Postpartum | 2021 |
| 928 | 2017 | Station B | Female | Droughtmaster | Postpartum | 2021 |
| 931 | 2017 | Station B | Female | Droughtmaster | Postpartum | 2021 |
| 932 | 2017 | Station B | Female | Droughtmaster | Postpartum | 2021 |
| 948 | 2016 | Station A | Male | Crossbred | NA | 2021 |
| 950 | 2019 | Station A | Male | Crossbred | NA | 2021 |
| 955 | 2019 | Station A | Male | Crossbred | NA | 2021 |
| 959 | 2017 | Station A | Female | Crossbred | Postpartum | 2021 |
| 960 | 2017 | Station A | Female | Crossbred | Postpartum | 2021 |
| 966 | 2017 | Station A | Female | Crossbred | Postpartum | 2021 |
| 972 | 2017 | Station A | Female | Crossbred | Postpartum | 2021 |
| 973 | 2017 | Station A | Female | Crossbred | Postpartum | 2021 |
| 17003 | 2017 | Station B | Female | Droughtmaster | Prepubertal | 2018 |
| 17009 | 2017 | Station B | Female | Droughtmaster | Cycling | 2018 |
| 17021 | 2017 | Station B | Female | Droughtmaster | Pregnant | 2018 |
| 17031 | 2017 | Station B | Female | Droughtmaster | Pregnant | 2018 |
| 17033 | 2017 | Station B | Female | Droughtmaster | Prepubertal | 2018 |
| 17037 | 2017 | Station B | Female | Droughtmaster | Prepubertal | 2018 |
| 17049 | 2017 | Station B | Female | Droughtmaster | Pregnant | 2018 |
| 17059 | 2017 | Station B | Female | Droughtmaster | Prepubertal | 2018 |
| 17065 | 2017 | Station B | Female | Droughtmaster | Cycling | 2018 |
| 17067 | 2017 | Station B | Female | Droughtmaster | Prepubertal | 2018 |
| 17087 | 2017 | Station B | Female | Droughtmaster | Cycling | 2018 |

Appendix 3: Sequence data generated for each sample using Oxford Nanopore Technologies long-read adaptive sequencing.

| **ID** | **Reads generated (in million)** | **Passed bases (Gb)** | **N50 (Kb)** | **Reads base-called** | **Total reads base-called (in million)** | **Total base-called bases (bp)** |
| --- | --- | --- | --- | --- | --- | --- |
| 264 | 4.33 | 5.69 | 2.54 | 4256014 | 4.26 | 5,626,437,843 |
| 265 | 2.87 | 8.41 | 5.15 | 2696940 | 2.70 | 8,062,422,806 |
| 267 | 3.43 | 14.01 | 6.81 | 3308828 | 3.31 | 13,773,841,645 |
| 270 | 5.03 | 7.43 | 2.23 | 4782974 | 4.78 | 7,112,062,770 |
| 271 | 1.26 | 1.49 | 1.98 | 1216056 | 1.22 | 1,449,633,527 |
| 272 | 3.78 | 3.49 | 5.48 | 3627606 | 3.63 | 3,399,566,185 |
| 273 | 2.22 | 1.97 | 1.07 | 6800103 | 6.80 | 6,818,651,285 |
| 277 | 1.17 | 1.15 | 1.53 | 1071686 | 1.07 | 1,112,453,627 |
| 278 | 3.57 | 6.14 | 2.9 | 3229467 | 3.23 | 5,796,997,292 |
| 280 | 4.67 | 4.37 | 1.59 | 4538184 | 4.54 | 4,260,892,668 |
| 294 | 3.207 | 2.183 | 0.661 | 3127610 | 3.13 | 2,141,025,345 |
| 295 | 15.37 | 6.85 | 0.5425 | 14563772 | 14.56 | 6,646,512,202 |
| 299 | 6.63 | 4.04 | 0.813 | 6339512 | 6.34 | 3,911,285,042 |
| 918 | 2.8 | 5.59 | 4.44 | 2571943 | 2.57 | 5,142,421,965 |
| 924 | 5.05 | 8.79 | 3.42 | 4965358 | 4.97 | 8,728,440,783 |
| 928 | 1.21 | 1.43 | 2.23 | 1161242 | 1.16 | 1,413,495,171 |
| 931 | 3.28 | 6.85 | 4.71 | 3222899 | 3.22 | 6,776,105,961 |
| 932 | 3.52 | 5.75 | 4.38 | 3465775 | 3.47 | 5,676,725,935 |
| 948 | 2.02 | 3.1 | 3.4 | 1933987 | 1.93 | 2,998,917,106 |
| 950 | 7.13 | 5.54 | 1.11 | 6916642 | 6.92 | 5,426,872,330 |
| 955 | 8.05 | 4.65 | 0.745 | 7620335 | 7.62 | 4,525,969,055 |
| 959 | 3.02 | 7.9 | 5.78 | 2960447 | 2.96 | 7,796,452,739 |
| 960 | 2.33 | 7.82 | 7.25 | 2276362 | 2.28 | 7,686,550,775 |
| 966 | 1.13 | 6.57 | 13.05 | 1110608 | 1.11 | 6,542,377,207 |
| 972 | 3.47 | 6.76 | 5.48 | 3366644 | 3.37 | 6,628,685,179 |
| 973 | 2.8 | 7.72 | 5.49 | 2735418 | 2.74 | 7,549,041,352 |
| 17003 | 3.32 | 10.73 | 5.41 | 3252463 | 3.25 | 10,652,335,222 |
| 17009 | 2.15 | 4.81 | 3.39 | 2112057 | 2.11 | 4,743,936,187 |
| 17021 | 2.34 | 8.28 | 6.07 | 2294822 | 2.29 | 8,216,926,124 |
| 17031 | 1.74 | 4.09 | 4.16 | 1580397 | 1.58 | 3,885,665,562 |
| 17033 | 5.35 | 12.12 | 3.38 | 5245707 | 5.25 | 12,031,109,522 |
| 17037 | 295 | 10.79 | 5.67 | 2929015 | 2.93 | 10,701,083,292 |
| 17049 | 2.53 | 8.17 | 5.2 | 2461945 | 2.46 | 8,016,352,126 |
| 17059 | 3.7 | 8.05 | 3.46 | 3583231 | 3.58 | 7,924,731,371 |
| 17065 | 6.6 | 9.41 | 2.28 | 6430150 | 6.43 | 9,302,677,341 |
| 17067 | 4.53 | 7.22 | 2.4 | 4386551 | 4.39 | 7,072,251,481 |
| 17087 | 5.03 | 3.68 | 0.79 | 4890047 | 4.89 | 3,514,500,050 |

Appendix 4: The proportions of sequence data belong to cattle host and metagenome

| **ID** | **Total bovine reads** | **Total bovine bases (bp)** | **Percentage of metagenome (%)** | **Coverage for 2Mb microbial genome** |
| --- | --- | --- | --- | --- |
| 264 | 198,717 | 284,828,450 | 5.06232288 | 142.41 |
| 265 | 329,312 | 506,067,731 | 6.27686916 | 253.03 |
| 267 | 221,969 | 459,335,808 | 3.33484165 | 229.67 |
| 270 | 818,758 | 740,929,032 | 10.4179203 | 370.46 |
| 271 | 111,984 | 82,701,340 | 5.70498257 | 41.35 |
| 272 | 346,691 | 234,253,417 | 6.89068558 | 117.13 |
| 273 | 757,812 | 728,681,473 | 10.6865924 | 364.34 |
| 277 | 193,235 | 158,238,098 | 14.2242422 | 79.12 |
| 278 | 389,348 | 361,149,721 | 6.22994462 | 180.57 |
| 280 | 334,384 | 355,679,675 | 8.34753895 | 177.84 |
| 294 | 235,026 | 265,431,706 | 12.3974107 | 132.72 |
| 295 | 2,076,438 | 1,158,545,958 | 17.4308859 | 579.27 |
| 299 | 954,090 | 611,712,398 | 15.6396783 | 305.86 |
| 918 | 105,657 | 204,420,486 | 3.97517915 | 102.21 |
| 924 | 160,842 | 227,173,917 | 2.60268612 | 113.59 |
| 928 | 142,568 | 126,813,925 | 8.97165605 | 63.41 |
| 931 | 154,410 | 282,128,443 | 4.16357779 | 141.06 |
| 932 | 199,205 | 226,870,603 | 3.99650442 | 113.44 |
| 948 | 274,129 | 410,384,119 | 13.6844102 | 205.19 |
| 950 | 897,759 | 1,196,605,124 | 22.0496273 | 598.30 |
| 955 | 1,089,832 | 714,175,506 | 15.7795048 | 357.09 |
| 959 | 139,579 | 191,630,555 | 2.45791979 | 95.82 |
| 960 | 130,383 | 307,588,680 | 4.00164767 | 153.79 |
| 966 | 43,016 | 155,786,162 | 2.38118588 | 77.89 |
| 972 | 246,430 | 267,469,799 | 4.03503548 | 133.73 |
| 973 | 166,762 | 278,007,482 | 3.68268591 | 139.00 |
| 17003 | 211,090 | 405,155,227 | 3.80344045 | 202.58 |
| 17009 | 134,913 | 207,829,081 | 4.38094175 | 103.91 |
| 17021 | 164,792 | 438,341,709 | 5.33461908 | 219.17 |
| 17031 | 245,697 | 342,723,459 | 8.82019962 | 171.36 |
| 17033 | 447,314 | 554,220,306 | 4.60656023 | 277.11 |
| 17037 | 166,833 | 345,933,903 | 3.23269985 | 172.97 |
| 17049 | 171,286 | 358,988,027 | 4.47819683 | 179.49 |
| 17059 | 362,737 | 437,934,741 | 5.52617774 | 218.97 |
| 17065 | 705,736 | 765,078,164 | 8.22427927 | 382.54 |
| 17067 | 484,867 | 436,845,873 | 6.17689959 | 218.42 |
| 17087 | 334,586 | 630,551,102 | 17.9414168 | 315.28 |

Appendix 5: Sequence data after quality filtering

| **ID** | **Total reads post QC** | **Total bases post QC (bp)** | **Coverage for 2MB genome** |
| --- | --- | --- | --- |
| 264 | 197,885 | 281,040,314 | 140.52 |
| 265 | 329,046 | 502,591,238 | 251.30 |
| 267 | 221,771 | 457,162,391 | 228.58 |
| 270 | 818,128 | 730,999,594 | 365.50 |
| 271 | 111,731 | 80,963,301 | 40.48 |
| 272 | 345,867 | 228,552,926 | 114.28 |
| 273 | 756,603 | 719,129,440 | 359.56 |
| 277 | 192,917 | 155,219,025 | 77.61 |
| 278 | 388,536 | 356,065,595 | 178.03 |
| 280 | 333,532 | 349,882,057 | 174.94 |
| 294 | 233,918 | 260,563,628 | 130.28 |
| 295 | 2,068,816 | 1,117,855,029 | 558.93 |
| 299 | 950,537 | 594,180,229 | 297.09 |
| 918 | 105,374 | 202,754,267 | 101.38 |
| 924 | 159,830 | 224,679,825 | 112.34 |
| 928 | 142,174 | 122,557,515 | 61.28 |
| 931 | 153,916 | 279,434,776 | 139.72 |
| 932 | 198,786 | 221,875,849 | 110.94 |
| 948 | 273,494 | 405,245,964 | 202.62 |
| 950 | 895,282 | 1,174,201,668 | 587.10 |
| 955 | 1,086,302 | 692,856,934 | 346.43 |
| 959 | 139,083 | 189,557,630 | 94.78 |
| 960 | 130,235 | 306,254,282 | 153.13 |
| 966 | 42,964 | 155,540,400 | 77.77 |
| 972 | 245,641 | 264,076,042 | 132.04 |
| 973 | 166,562 | 276,141,091 | 138.07 |
| 17003 | 210,710 | 400,628,961 | 200.31 |
| 17009 | 134,486 | 204,098,344 | 102.05 |
| 17021 | 164,155 | 433,871,199 | 216.94 |
| 17031 | 245,251 | 336,837,297 | 168.42 |
| 17033 | 446,387 | 540,244,118 | 270.12 |
| 17037 | 166,458 | 343,127,135 | 171.56 |
| 17049 | 170,873 | 355,759,460 | 177.88 |
| 17059 | 362,397 | 428,763,751 | 214.38 |
| 17065 | 703,371 | 745,011,644 | 372.51 |
| 17067 | 484,020 | 423,604,290 | 211.80 |
| 17087 | 333,965 | 624,683,739 | 312.34 |


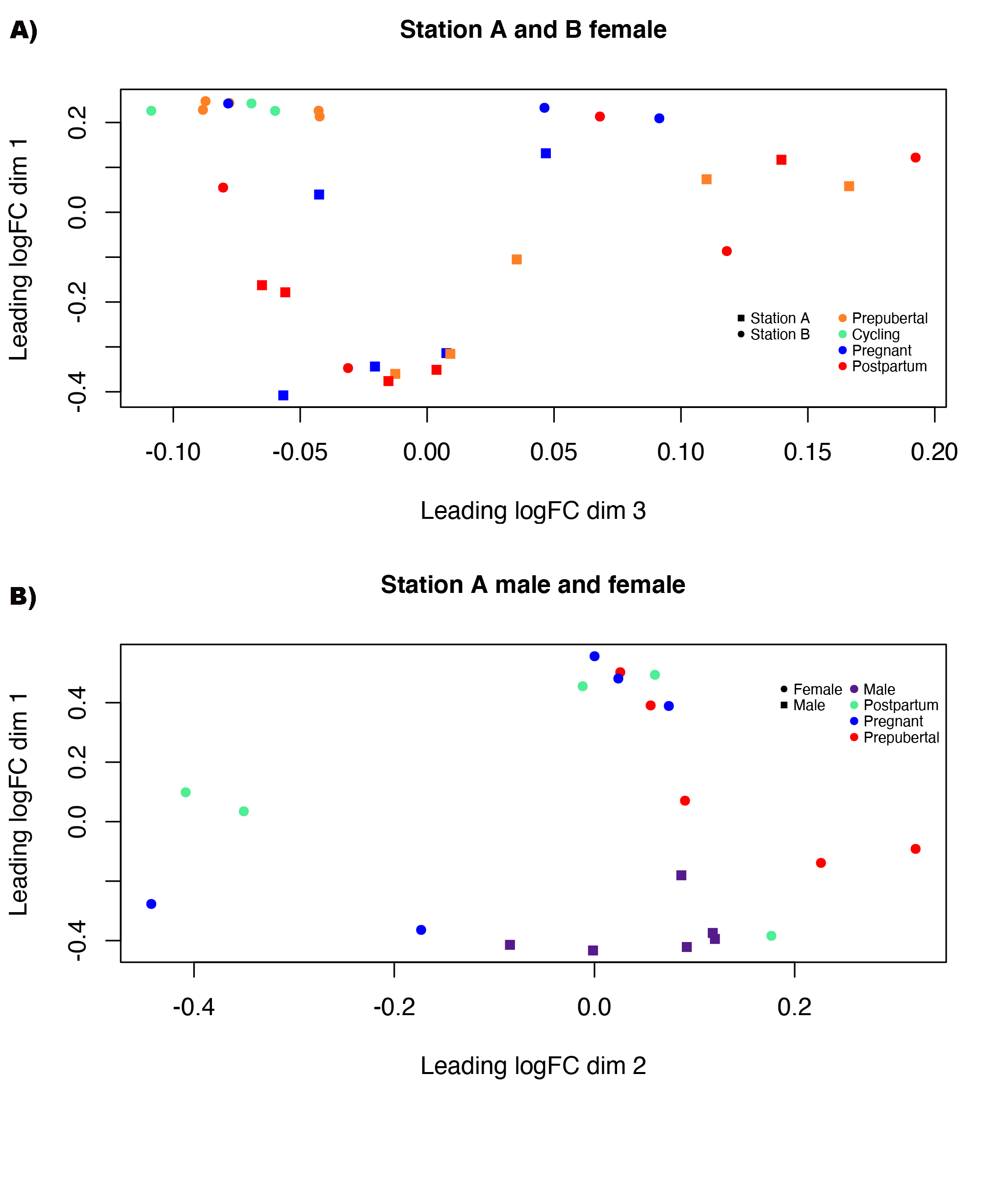


Appendix 6: Multidimensional scaling (MDS) analysis of the dissimilarity based on the leading log_2_ fold change of the functional annotations of the reproductive metagenomes between (A) female from Station A and Station B and between (B) male and female from Station A.
